# Drivers of demographic decline across the annual cycle of a threatened migratory bird

**DOI:** 10.1038/s41598-018-25633-z

**Published:** 2018-05-09

**Authors:** Scott Wilson, James F. Saracco, Richard Krikun, D. T. Tyler Flockhart, Christine M. Godwin, Kenneth R. Foster

**Affiliations:** 1 Wildlife Research Division, Environment Canada, National Wildlife Research Centre, 1125 Colonel by Drive, Ottawa, ON K1A 0H3 Canada; 2 The Institute for Bird Populations, PO Box 1346, Point Reyes Station, CA 94956 USA; 3 Lesser Slave Lake Bird Observatory, Box 1076, Slave Lake, AB T0G2A0 Canada; 4 University of Maryland Center for Environmental Science, Appalachian Laboratory, 301 Braddock Road, Frostburg, MD 21532 USA; 5 Owl Moon Environmental Inc., 324 Killdeer Way, Fort McMurray, Alberta, T9K 0R3 Canada

## Abstract

Migratory species are rapidly declining but we rarely know which periods of the annual cycle are limiting for most species. This knowledge is needed to effectively allocate conservation resources to the periods of the annual cycle that best promote species recovery. We examined demographic trends and response to human footprint for Canada warblers (*Cardellina canadensis*), a threatened Neotropical migrant, using range-wide data (1993–2016) from the Monitoring Avian Productivity and Survivorship (MAPS) program on the breeding grounds. Declines in abundance were steepest in the eastern breeding region, followed by the western region. Breeding productivity did not decline in any region. In contrast, we observed declining recruitment in all regions, low apparent survival in the east and west, and a decline in apparent survival in the east. Abundance declined with increasing disturbance around MAPS stations. Between 1993 and 2009, the human footprint index on the breeding range increased by 0.11% in contrast to a 14% increase on the wintering range. Landscape-scale disturbance on the breeding grounds may influence abundance in some regions; however, the observed trends in demography and footprint suggests limitation during the non-breeding period as the likely driver of overall declines, particularly for eastern populations.

## Introduction

Worldwide, many populations of migratory animals are undergoing rapid declines^1,2^. These declines not only prompt concern for the general loss of biodiversity per se but also for how such losses might influence ecological function^3^. Identifying management action to reverse declines of migratory species is complex because individuals move among distinct geopolitical regions throughout the annual cycle, and may experience a different suite of threats during each stage^4,5^. General factors underlying declines of migratory taxa include land-use change, barriers to migration, overexploitation and climate change^1,6,7^. However, the specific mechanisms by which these factors influence populations and when in the annual cycle they are most influential will differ among species^5,8,9^. Accordingly, for many migratory species we have yet to discern whether range-wide declines are primarily due to a change in fecundity, juvenile recruitment or adult survival, let alone the specific factors underlying a change in these vital rates. Knowledge of the geographic locations and periods of the year that have the greatest impact on populations can aid the development of conservation strategies that most effectively promote species recovery while avoiding expenditure of limited resources on strategies that have little ability to reverse declines^7,9^.

Population declines are notable for many species of songbirds that move annually between breeding grounds in the temperate zone and wintering grounds in the tropics^10,11^. Effective conservation action to reverse declines within this group is aided by two key pieces of information. First, we need a knowledge of migratory connectivity; where do particular breeding populations of a species move during the non-breeding period and vice versa^4^? Our understanding of migratory connectivity has improved substantially in recent years owing to advances in the use of isotopic analysis, genetics and the application of new tracking technology^12–14^. Second, we require an understanding of the anthropogenic and/or natural factors that have changed in the breeding and/or non-breeding environments, and how these changes have impacted demography and abundance. Local studies of demography and population change of Neotropical migrant birds have helped identify mechanisms that limit and regulate populations during breeding^15–17^ and non-breeding periods^18,19^. However, most studies have been conducted over small spatial scales and typically do not reveal why populations are declining across broad regions; addressing this question requires studies at spatial scales covering the distribution of a species. Research at such broad scales is challenging, but the combination of long-term and spatially extensive datasets (e.g. Monitoring Avian Productivity and Survivorship Program^20^; North American Breeding Bird Survey^11^) with knowledge of the change in factors such as land use or climate can allow us to identify the mechanisms that influence annual variability or long-term trends of populations across broad spatial scales^6,7,21^.

Songbird population declines have been particularly severe for some Neotropical migratory species that breed in the boreal and eastern hardwood forests of North America and overwinter in the Andean mountains of northwestern South America^11,22^. For these species we still have a limited understanding of the relative importance of threats during the breeding and non-breeding periods or what changes in the demographic rates underlie population declines. González-Prieto *et al*. (ref.^23^) recently identified fine-scale migratory connectivity for one of these declining species, the Canada warbler (*Cardellina canadensis*). They showed that individuals over-wintering along the eastern slopes of the Andes were primarily from eastern portions of the breeding range, while individuals that over-wintered further west in the Magdalena and Cauca basins were primarily from western and central breeding populations. Forest loss has been extensive in the northern Andes in recent years^24^ raising speculation that declines in Canada warbler abundance may be driven by conditions on the non-breeding grounds.

**Figure 1:**
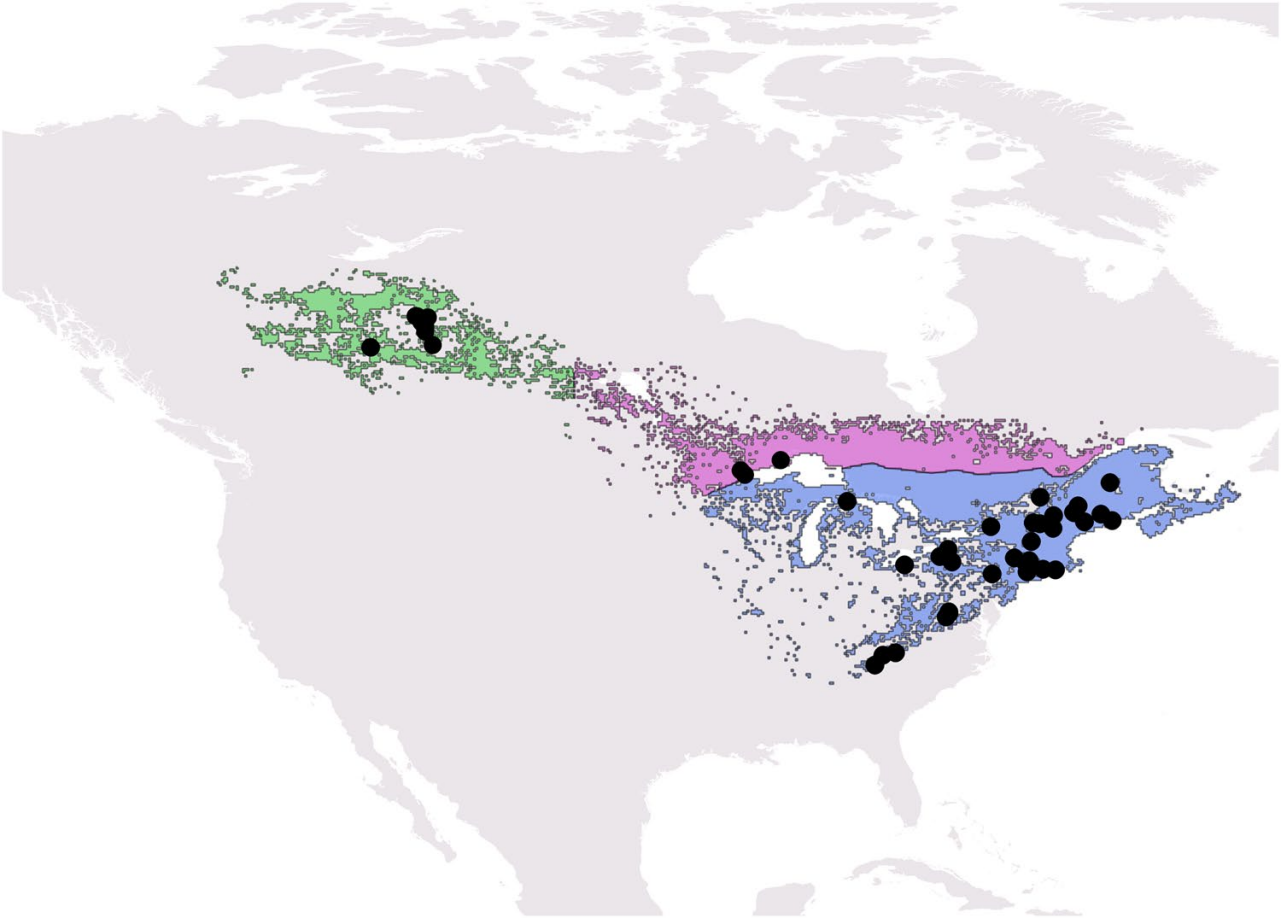
Canada warbler breeding distribution showing the western (green), central (purple) and eastern (blue) breeding regions specified in this analysis. Black circles show MAPS station locations. Distribution maps are based on eBird spatio-temporal exploratory models^52–54^ and were created using QGIS Version 2.18^59^.

We used a Bayesian approach to analyze temporal trends in demography and response to anthropogenic footprint by Canada warblers across the species breeding range. Using 24 years of data (1993–2016) collected from the Monitoring Avian Productivity and Survivorship Program^25^ (MAPS), we first asked how adult breeding density, breeding productivity, adult apparent survival and recruitment varied over time in west, central and eastern portions of the breeding range (Fig. 1). We identified these three regions in part based on the connectivity results of González-Prieto *et al*. (ref.^23^, see Methods). Our aim was to identify whether any change in demographic rates within regions were more consistent with a breeding ground or a non-breeding ground influence on population declines. Temporal declines in breeding productivity would suggest that factors on the breeding grounds have contributed to declines in abundance. In contrast, temporal declines in adult survival or recruitment would more likely be driven by threats during the non-breeding period, as previous research indicates that the vast majority of annual mortality for Neotropical migrants occurs during the non-breeding season^26–28^. Our second objective examined how the extent of breeding ground human footprint^29^, a composite measure of anthropogenic impact, influenced adult breeding density, breeding productivity, adult apparent survival and residency probability. Finally, we estimated the changes in human footprint between 1993 and 2009 for western, central and eastern regions of the breeding range and for the entire wintering range. For the latter, we also estimated the change in human footprint in the Magdalena/Cauca basins and the eastern slopes of the Andes in Colombia since Canada warblers over-wintering in these regions primarily bred in west/central and eastern parts of the breeding range, respectively^23^.

## Results

### Temporal trends in abundance and demography

**Figure 2:**
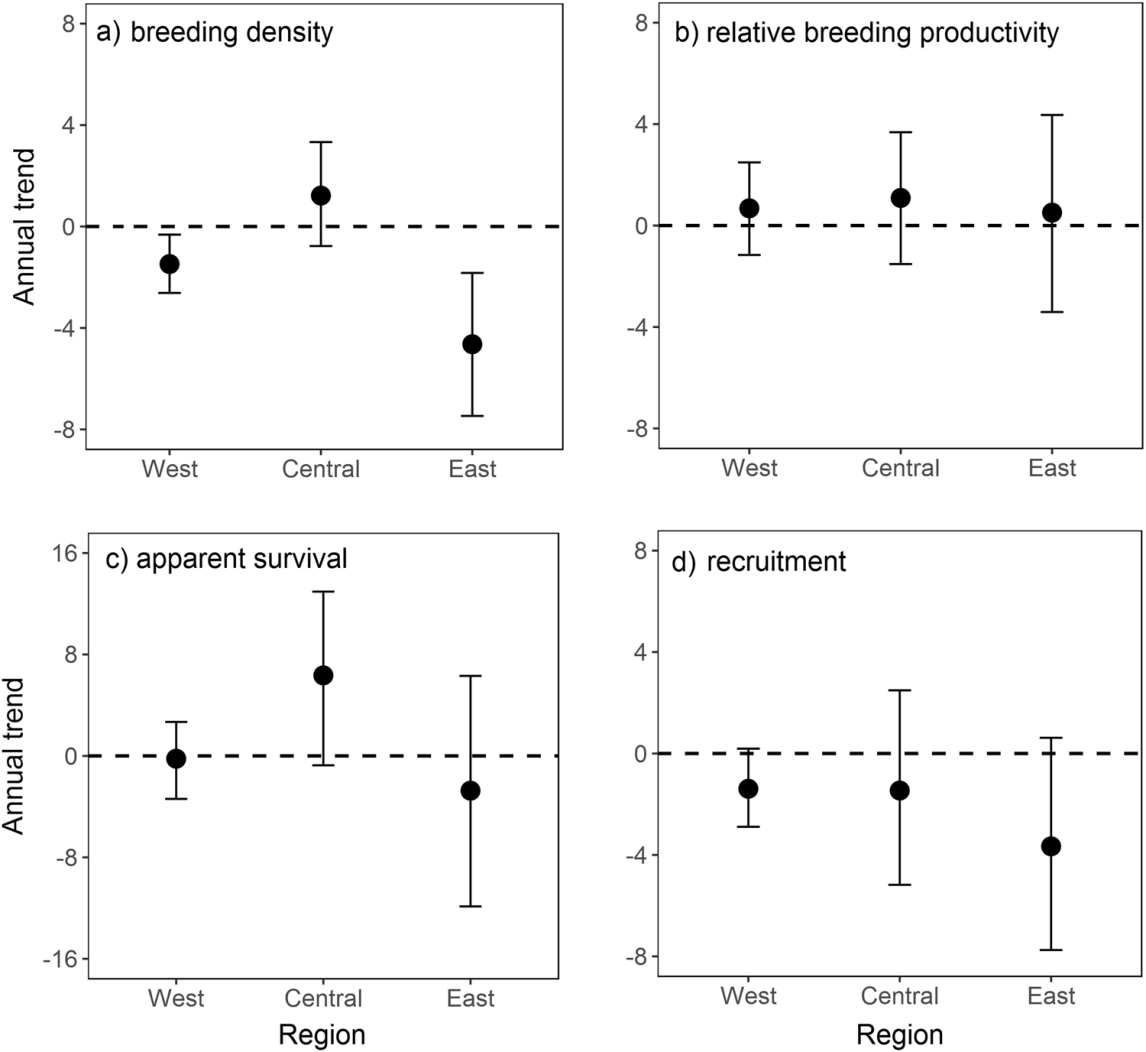
Annual trend coefficients and 90% credible intervals for breeding density, relative breeding productivity, apparent annual survival of breeders and annual recruitment of Canada warblers. Upper credible intervals below 0 indicate a greater than 90% probability of a decline over the course of the study (1993–2016). Note the different scale for the y-axis for apparent survival.

The strongest declines in abundance were in the eastern region averaging −4.64% per year with a >99% probability of a decline (Fig. 2, Table S1). Negative trends were milder in the western region at −1.48% per year and a 98% probability of decline. Trend coefficients were positive for the central region at 1.21% per year and an 87% probability of increase (Fig. 2, Table S1). There was no evidence for declines over time in relative breeding productivity, estimated as the probability that an individual captured was a juvenile rather than an adult (Fig. 2, Table S1). In fact, annual trend coefficients for all regions were positive. Mean relative breeding productivity was similar among the central (0.43, 90% CI: 0.27, 0.60), western (0.35, 90% CI: 0.28, 0.41) and eastern regions (0.32, 90% CI: 0.26, 0.39).

Apparent survival probabilities were higher for males than females (${\hat{{\rm{\beta }}}}_{{\rm{\varphi }},2}=0.58$, 90% CI: 0.08, 1.07) and were higher in the central region (Table 1). Apparent survival probabilities for females and males were particularly low (<0.5) in the western and eastern regions (Table 1). The eastern region had some evidence for a negative trend in apparent survival with a median annual decline of −2.74% per year but credible intervals were wide with only 70% of the posterior mass below 0 (Fig. 2, Table S1). The strongest trend in apparent survival for any region in either direction was the positive trend in the central region at 6.35% per year although with low certainty on the estimate and based only on 3 stations (Fig. 2, Table S1). Recapture probability averaged 0.56 (95% CI: 0.42, 0.68) for males and 0.27 (95% CI: 0.14, 0.41) for females (${\hat{{\rm{\beta }}}}_{{\rm{pr}},2}=1.25$, 95% CI: 0.60, 1.88). Residency probability, defined as the probability that an individual captured at the site remained at the site during the breeding season, was more than twice as high in the western region compared to the central and eastern regions (Table 1) and averaged 0.09–0.10 higher for males than females (${\hat{{\rm{\beta }}}}_{{\rm{\pi }},2}=0.42$, 95% CI: −0.24, 1.08).

**Table 1:** Estimated mean apparent adult survival and residency probability of male and female Canada warblers from western, central and eastern regions of the breeding range based on data from the Monitoring Avian Productivity and Survivorship program. Values shown include the mean and 90% credible interval from the posterior distribution.

Group	Apparent survival probability	Residency probability
West female	0.31 (0.18, 0.43)	0.58 (0.39, 0.77)
Central female	0.46 (0.28, 0.66)	0.28 (0.12, 0.51)
East female	0.30 (0.15, 0.46)	0.23 (0.11, 0.36)
West male	0.45 (0.35, 0.55)	0.67 (0.52, 0.83)
Central male	0.61 (0.44, 0.77)	0.38 (0.19, 0.61)
East male	0.44 (0.30, 0.58)	0.32 (0.20, 0.43)

Derived trend estimates for recruitment were more negative than trends for adult survival. The median trend coefficients were negative in all three regions although effect sizes differed with an average −3.66% annual decline in the east, −1.46% in the central region and −1.39% in the west (Fig. 1, Table S1). As with apparent survival, the outer tails on the credible intervals were wide although our certainty for a decline in recruitment was relatively high for both the east (91%) and west (92%).

### Response to anthropogenic footprint

The human footprint index at 1 km and 10 km radii scales around MAPS stations was lower for the 3 stations in the central region compared to the 23 stations in the western region and the 33 stations in the eastern region, the latter two of which were similar (Table 2). Between 1993 and 2009, the average footprint across all stations increased by 16.5% and 20.1% at 1 km and 10 km radius scales, respectively. This increase over time was greater for the western stations (34.3% at 1 km, 56.9% at 10 km) than the central (5.1% at 1 km, 1.2% at 10 km) and eastern stations (3.0% at 1 km, 4.9% at 10 km).

**Table 2:** Human footprint indices (mean and 90% CI) in 1993 and 2009 at 1 km and 10 km radii around MAPS stations in west, central and eastern regions of the Canada warbler breeding range. Values of the Human Footprint Index range from 0 (no footprint) to 50 (high footprint).

Region	1 km radius		10 km radius	
	1993	2009	1993	2009
West	10.72 (7.39, 14.06)	14.40 (11.20, 17.60)	6.60 (4.71, 8.48)	10.35 (8.06, 12.65)
Central	3.28 (1.94, 4.61)	3.44 (1.89, 4.99)	3.20 (1.60, 4.80)	3.23 (1.60, 4.87)
Eastern	9.61 (7.41, 11.81)	9.90 (7.76, 12.04)	10.82 (8.77, 12.86)	11.35 (9.23, 13.46)
Total	9.72 (7.92, 11.53)	11.33 (9.51, 13.14)	8.78 (7.34, 10.23)	10.55 (9.03 12.07)

Across the breeding range, the footprint index increased by 0.11% between 1993 and 2009 with a declining footprint in the western and central region, and a slight increase in the footprint in the eastern region (Table 3). Thus, increasing trends in footprint were much higher around the MAPS stations in this analysis than they were for the range as a whole. Across the winter range, the footprint index increased by 14.0% between 1993 and 2009. The average footprint was higher in the Cauca and Magdalena basins than in the Piedmont region but the rate of change was more than twice as high in the latter (Table 3).

**Table 3:** Human footprint indices in 1993 and 2009 for the breeding and wintering ranges including the western, central and eastern breeding regions and the Cauca-Magdalena and Piedmont wintering regions in Colombia.

Region	1993	2009	% change
Western	1.354	1.122	−17.0
Central	1.890	1.760	−6.9
Eastern	8.922	9.083	2.3
Breeding range	5.446	5.451	0.11
Cauca/Magdalena	8.486	9.434	11.2
Piedmont	4.673	5.728	22.6
Wintering range	5.434	6.203	14.0

The breeding density and residency probability of Canada warblers was negatively influenced by the extent of human footprint at 1 km and 10 km radii scales around stations (Table 4). There were no negative effects of footprint on apparent adult survival at either scale. The coefficient for the effect of footprint on relative breeding productivity was positive and similar at the two scales (Table 4).

**Table 4:** Model coefficients showing the influence of disturbance (based on human footprint indices) at 1 km and 10 km radius scales around MAPS stations on breeding abundance, productivity, apparent survival and residency probability of Canada warblers.

Variable	Spatial scale	
	1 km	10 km
Breeding abundance	−0.66 (−1.00, −0.33) >0.999	−0.73 (−1.06, −0.39) >0.999
Productivity	0.16 (−0.002, 0.32) 0.05	0.19 (−0.002, 0.42) 0.05
Apparent survival	0.03 (−0.33, 0.44) 0.45	0.09 (−0.24, 0.48) 0.32
Residency probability	−0.46 (−0.80, −0.12) 0.98	−0.40 (−0.77, −0.10) 0.97

Values shown include the mean and 90% credible interval from the posterior distribution with the probability of a negative effect underneath.

## Discussion

North American Breeding Bird Survey (BBS) data indicate a significant decline of Canada warblers at the continental scale since the early 1990s but with regional variation^11,22^. Population declines tend to be strongest in the eastern hardwood and boreal regions, moderate in the western boreal forest and only slight in central parts of the range (e.g. Minnesota, Manitoba). Our results using MAPS data from across the breeding range supports this pattern. We found the strongest declines in abundance in the eastern region, milder declines in the western region and a slight positive trend in the central region. Similar patterns in the trends across two independent data sets provides an indication of the true regional trends in Canada warbler breeding populations.

We found positive trend coefficients for relative breeding productivity across all regions suggesting that the Canada warbler population decline is unlikely to be due to any changes in the breeding or non-breeding environments that have impacted breeding success. While the certainty in demographic rates tended to be low, the evidence from our analyses suggest that declines in abundance at MAPS stations in the eastern and western regions of the range were more likely related to 1) declines in recruitment, 2) low average apparent survival in both regions and 3) a potential negative trend in apparent survival in the eastern region. Trends in abundance at finer scales can be driven by movement, however, this becomes less influential for population dynamics as the scale of the analysis increases^17,30^. Movement was incorporated in our analysis both through the estimate of recruitment, which includes immigrants, and apparent survival, which includes permanent emigration. Because we had a large sample size of individuals and stations spread across a broad spatial area in the east and west, it is very unlikely that a consistent change in movement across all stations contributed to the mean estimates and trends in demography in those two regions. However, caution is needed in interpreting the trends in abundance, survival and recruitment from the central region because 95% of marked individuals were from two stations and any local patterns in movement at those stations would strongly influence our regional estimates.

Our method for estimating recruitment as a derived parameter incorporated productivity, juvenile survival through the non-breeding season and their subsequent settlement on the breeding grounds in their first breeding season. Recruitment also includes adult immigrants that may have bred previously but, as described above, it is unlikely that there were consistent trends in immigration across large spatial scales and therefore the recruitment estimate in the eastern and western regions should almost entirely represent the recruitment of new individuals in their second year. Our independent estimation of relative breeding productivity showed that there was no evidence for a decline in breeding success and this includes the post-fledging period given the estimation of productivity as a probability of catching juveniles relative to the total numbers of juveniles and adults. This finding indicates that declines in recruitment were more likely due to factors affecting juveniles after they departed the natal area in late summer. This period includes a very short post-breeding window prior to fall migration and the approximately 8 to 9-month period between the onset of fall migration and the start of the subsequent breeding season. Research on differences in juvenile survival between the breeding and non-breeding seasons is limited but studies of adult survival for other Neotropical migrant songbirds have shown that most annual mortality occurs during the non-breeding period^26–28,31^. Similar patterns have been observed for other avian taxa including shorebirds and raptors^32,33^. Most of these studies indicate that the migratory periods are when most annual mortality occurs while monthly survival rates are often similar between breeding and stationary non-breeding (i.e. winter) periods. Yet, because Neotropical migrants spend 2–3 times longer on the wintering than the breeding grounds, absolute mortality is typically higher during winter. In addition, stresses experienced during a stationary period of the annual cycle may carry over and manifest during a transitory or subsequent stationary period. For example, poor winter habitat quality may ultimately influence individuals during the more energetically demanding spring migration^31,34^. Seasonal survival estimates are typically derived from small scale studies in higher quality habitats that are feasible for long-term demographic research. As a consequence, we have less knowledge on how differing rates of habitat loss across the annual cycle influence seasonal survival.

Juvenile survival is one of the least understood demographic rates presenting challenges in how best to implement conservation measures for cases where declining recruitment of juveniles may be an issue affecting population viability^9^. Studies during the migratory and winter periods point to juvenile birds suffering lower body condition relative to adults in the same environment, likely due to a combination of inexperience in selecting optimal sites and competition from adults^19,35,36^. In Jamaica for example, experimental studies on American redstarts (*Setophaga ruticilla*) showed that the removal of adults from high quality winter territories led to upgrading by juveniles with subsequent improvement in their condition^37^ (see also ref.^35^). Such effects of competition on juveniles may be amplified in environments where habitat loss results in increasing habitat limitation over time (e.g. ref.^38^).

Our analysis on the change in human footprint between 1993 and 2009 showed only a 0.11% increase in footprint across the breeding range but a 14% increase across the winter range. That increase was particularly high in the eastern slopes of the Andes (23%) where over-wintering individuals are primarily from eastern and southern portions of the breeding range^23^. Moreover, the breeding range is approximately 5.3 times the size of the winter range (estimate of 1,947,887 km^2^ versus 365,381 km^2^ for the breeding and winter ranges, respectively, Fig. S1). Although space requirements for individuals and their tolerance to anthropogenic disturbance may not be the same between the two periods, the difference in range size suggests a likely greater impact of a given area of habitat loss in winter. A broad scale study of population trends of European migratory birds showed that species with larger breeding than non-breeding ranges (i.e. low migratory dispersion) were more likely to be declining than species with larger non-breeding than breeding ranges^39^. Canada warblers are only one of a number of declining Neotropical migrants that overwinter in the Andes of northwest South America and show low migratory dispersion; others include cerulean warbler (*Setophaga cerulea*), Eastern wood-pewee (*Contopus virens*), and olive-sided flycatcher (*Contopus cooperi*)^11,22^. While it is clear that there has been considerable anthropogenic change on the wintering grounds of these species, the extent to which they are also experiencing increased habitat loss and/or other threats at key stopover sites along the spring and fall migratory routes is less clear. Canada warblers are a circum-gulf migrant with mixing of individuals from across the breeding range during passage through the southern portion of the migratory route in the Darién region of Panama and northern Colombia^40^. Field research aimed at understanding the impacts of landscape change along the migratory routes and wintering grounds on condition and monthly survival of juveniles and adults would be a valuable area of future study.

Our finding that both abundance and residency probability were negatively affected by the extent of anthropogenic footprint around stations indicates that development on the breeding grounds could impact the species in some areas. Footprint indices were higher around MAPS stations than for the breeding range as a whole, which would be expected because road access is required and the stations tend to be closer to developed areas. Moreover, in the western region, there were positive trends in footprint around MAPS stations despite an overall negative trend in footprint for the western region. This contrast is due primarily to the fact that many of the western stations were established to examine the impacts of natural resource development and subsequent restoration efforts on the boreal bird community in Alberta^41^. Thus, declines in breeding settlement due to increasing footprint may have contributed to declines at stations in that region, although Breeding Bird Survey data from Alberta also indicate broader scale declines of Canada warblers in the province^22^. Increasing footprint around stations is not an explanation for declines in the eastern region where there was less than a 0.3% annual increase in footprint over the time period of this study. Using data from the Boreal Avian Modeling project, Haché *et al*. (ref.^42^) found similar results with lower Canada warbler abundance in areas that had a higher proportion of agriculture and human development within 16 km of survey points.

Our measure of anthropogenic footprint does not explicitly include a change in forest cover related to forestry practice but would include changes to the landscape that accompany forestry activities (e.g. an increase in roads, buildings or lighting^29^). Thus, the higher levels of disturbance that resulted in lower abundance and residency probability in our study were more likely related to agricultural, urban, and natural resource development (e.g. oil and gas, mining). Remotely sensed estimates of forest cover change in the boreal forest between 1985 and 2010 indicate slight declines of about −3.2% and −4.4% for the Boreal Plains and Boreal Shield ecozones respectively^43^. Yet, understanding how Canada warblers are influenced by changes in forest cover due either to natural variation (e.g. fire, insect outbreaks) or forestry activities is complex because of the diversity of habitat types and successional stages used by the species throughout their range. In the western boreal forest, the abundance of territorial males was higher around unharvested than post-harvest stands, although conspecific attraction also had an influence on selection of stand types^44^. In hardwood forests of the northeastern US and along the southern edge of the range in the Appalachian Mountains, Canada warbler abundance can be positively influenced by forest management practices that create small patches of early successional habitat^45–48^.

## Conclusions

Data from broad-scale monitoring programs have long provided us with information on which migratory species are declining at distribution-wide scales. However, we often have a limited understanding of the cause of those declines and a key question is whether factors on the breeding or the non-breeding grounds have had a greater impact on species populations. Our range-wide and long-term analysis of multiple demographic rates suggests that while landscape-scale habitat loss on the breeding grounds may influence abundance in some regions, the non-breeding period is likely the primary source of the decline for Canada warbler populations. Not only were the patterns in the demographic rates consistent with a non-breeding season effect (i.e. low or declining survival and recruitment rather than breeding productivity) but the trend in anthropogenic development has been far greater on the wintering compared to the breeding range. To date, few studies have linked knowledge of migratory connectivity with analysis of full life cycle demography but the approach we have used could be applied to several other declining Neotropical migrants. Species with a more southern distribution in particular would have a high coverage under the MAPS program allowing for precise estimation of the demographic rates that underlie declining abundance.

## Methods

### Study species

The Canada warbler is listed as a threatened species under Canada’s Species-at-Risk Act, and is the focus of international conservation initiatives (e.g. www.partnersinflight.org). The Canada warbler breeding range includes much of the southern boreal forest of Canada and the northeastern US with the southward extent of their range in the Appalachian region reaching Georgia^48^. Preferred breeding habitat typically consists of a dense understory and complex ground cover within forested landscapes but the type of forest used to meet these requirements varies across the range^46,49,50^. Canada warblers primarily over-winter in northwest South America at mid-elevations (typically 1000–1800 m) in the Andean mountains^23,49^. Winter habitats frequently include a dense understory, but can otherwise be in cloud forest, early to mid-successional woodlands, shade-grown coffee plantations and shrubby forest edge^51^. Canada warblers arrive on the breeding grounds in mid to late May and typically depart the northern breeding grounds between late July and mid-August^49^.

### Canada warbler distribution maps

We used eBird spatiotemporal exploratory models^52–54^ to identify the breeding and wintering range of Canada warblers. These models estimate species occupancy and abundance at an 8.4*8.4 km resolution and at weekly intervals. We selected 5-week periods for the breeding (June 6–July 11) and wintering (Dec 26–Jan 30) ranges and merged the weekly raster layers to create a layer for the breeding and for the non-breeding ranges.

### Demographic Data

The Monitoring Avian Productivity and Survivorship (MAPS) program is a cooperative network of >1200 constant effort mist-netting stations operated during the breeding season (May through early August) and provides demographic data for more than 180 landbird species across North America^20,25,55^. We used MAPS data from 1993 to 2016 for this analysis. We defined three main breeding regions (west, central, east) in part based on previous research on migratory connectivity of Canada warblers^23^. In that study, individuals overwintering in the Piedmont region of Colombia primarily bred in eastern and southern portions of the breeding range, while those overwintering further west in the Magdalena and Cauca basins primarily bred in central and western regions of the breeding range. The location of our stations in the western and central regions would appear to include individuals overwintering in the Cauca basin and northern and southern regions of the Magdalena basin. Because of the broad geographic distance among the MAPS stations in Alberta and Ontario/Minnesota we consider these as western and central regions respectively with the division between the two regions at 102°W longitude (approximate mid-point between the stations in Alberta and Ontario/Minnesota). The eastern region included stations south of 47° latitude across the eastern US and the Canadian maritime provinces (Fig. 1).

MAPS stations sampled an area of about 20 ha and operated on a standard field protocol consisting of mist-netting for a 6-hr period on 6 to 9 days (dependent on the latitude of the station) in each breeding season (May-Aug). Banding was conducted once in each 10-day period (i.e., at approximate 10-day intervals). Upon capture, unmarked birds were sexed, aged, measured, assessed for breeding status and banded with a uniquely numbered band from the United States Geological Survey Bird Banding Laboratory^25^. Band numbers of recaptured birds were recorded, and the recaptured birds were processed for sex, age and breeding status. All birds were captured and banded following regulations and permits issued by the United States Geological Survey Bird Banding Laboratory and the Canadian Wildlife Service Bird Banding Office.

### Human Footprint Index

We investigated relationships between Canada warbler demography and anthropogenic development using the global terrestrial human footprint index^29,56^. This index provides a cumulative measure of human pressure on the environment and has been shown to be a strong predictor of ecological and conservation metrics^57,58^. The index is a composite of eight measures of human pressure: 1) the extent of built environments, 2) crop land, 3) pasture land, 4) human population density, 5) night-time lights, 6) railways, 7) roads, and 8) navigable waterways. These eight measures are individually weighted based on their relative levels of human pressure and summed to create a single standardized estimate^29^. The index varies from 0 (no footprint) to 50 (very high footprint) and is estimated at a 1 km resolution across all global terrestrial lands except Antarctica. For additional detail on the calculation of the human footprint index see refs^29,54^. We used QGIS Version 2.18^59^ to extract the value of the human footprint index in 1993 and 2009 at a 1 km radius (3.14 km^2^) and a 10 km radius (314 km^2^) circle around each of the MAPS stations. These two different radii were selected to examine whether the human footprint was more influential at local versus broader landscape scales. We aimed to use a value of human footprint that best represented the time period during which data was collected at each station. If a station was active in 1993 and 2009 we used the average value for the two periods (n = 6 stations). For all other stations we calculated the median survey year and selected the value for the year that was closest to this median year (1993: 16 stations, 2009: 37 stations). We also estimated the average human footprint in 1993 and 2009 for 1) the entire breeding range, 2) the entire wintering range, 3) each of the three breeding regions identified in this analysis and 4) the Magdalena/Cauca basins and eastern slopes (Piedmont) regions in Colombia based on ref.^23^.

### Statistical Analysis

Estimates of relative adult breeding density (hereafter ‘breeding density’) were based on stations where at least one juvenile or adult was captured over the 23-yr period resulting in data from 59 MAPS stations, 483 station-years and 1557 banded individuals. A total of 875 adults (i.e. after hatch year) were marked at 23 stations in the western region, 262 adults at 4 stations in the central region and 420 adults at 32 stations in the eastern region. We modeled the annual count of adults (C_*k,s,t*_) (an index of breeding density) for region *k*, MAPS station *s* and year *t* as a Poisson random variable with mean λ_*k,s,t*_ defined as a log-linear relation to the predictor variables:

1 $$\mathrm{log}({{\rm{\lambda }}}_{k,s,t})={{\rm{\alpha }}}_{{{\rm{\lambda }}}_{k}}+{{\rm{\beta }}}_{{\rm{\lambda }},{1}_{k}}\ast t+{{\rm{\beta }}}_{{\rm{\lambda }},2}\ast {{\rm{HF}}}_{s}+{{\rm{\beta }}}_{{\rm{\lambda }},3}\ast {{\rm{EF}}}_{s}+{{\rm{\omega }}}_{{{\rm{\lambda }}}_{s}}+{{\rm{\varepsilon }}}_{{{\rm{\lambda }}}_{k,s,t}}$$

The model includes region-specific estimates for average abundance per MAPS station $({{\rm{\alpha }}}_{{{\rm{\lambda }}}_{k}})$ and log-linear population trends $({{\rm{\beta }}}_{{\rm{\lambda }},{1}_{k}})$. ${{\rm{\beta }}}_{{\rm{\lambda }},2}$ represents the effect of human footprint (HF) on station-level breeding density and was assumed to be a common relationship across all regions. Because of strong correlations for the footprint indices at 1 km and 10 km radii (r = 0.80, n = 59 stations) we estimated ${{\rm{\beta }}}_{{\rm{\lambda }},2}$ for the two scales separately. ${{\rm{\beta }}}_{{\rm{\lambda }},3}$ represents the effect of annual station effort (EF, number of mist-net hours) on estimates of breeding density. Our model also included station-level variance as a random variable $({{\rm{\omega }}}_{{{\rm{\lambda }}}_{s}})$ and count-level variability $({{\rm{\varepsilon }}}_{{{\rm{\lambda }}}_{k,s,t}})$, which helps accommodate over-dispersion expected in count data.

Analyses on relative breeding productivity were based on the same dataset as for breeding density but we removed 4 stations that only had juvenile (JUV) captures resulting in 2447 individuals at 55 stations. Total juvenile captures included 481, 167 and 242 individuals in the western, central and eastern regions respectively. We estimated relative breeding productivity as the probability of catching a juvenile bird given the total number of juveniles and adults at MAPS stations. This probability of catching a juvenile is frequently used as an index of productivity with MAPS data and thereby incorporates nesting success and post-hatch juvenile survival through the last MAPS sampling period, typically in late July^25^. We used the following equation to estimate productivity:

2 $$\begin{array}{c}{{\rm{JUV}}}_{k,s,t} \sim {\rm{binomial}}({p}_{k,s,t},{N}_{k,s,t})\\ {\rm{logit}}({p}_{k,s,t})={{\rm{\alpha }}}_{{p}_{k}}+{{\rm{\beta }}}_{p,{1}_{k}}\ast t+{{\rm{\beta }}}_{p,2}\ast {{\rm{HF}}}_{s}+{{\rm{\omega }}}_{{p}_{s}}\end{array}$$

where N_*k,s,t*_ is the total number of individuals captured in region *k*, MAPS station *s* and year *t*, and *p* is the probability that an individual captured in region *k*, MAPS station *s* and year *t* was a juvenile. This index of breeding productivity is estimated as a region-specific mean $({{\rm{\alpha }}}_{{p}_{k}})$ and temporal trend $({{\rm{\beta }}}_{p,{1}_{k}})$ with ${{\rm{\beta }}}_{p,2}$ again representing a common effect of human footprint at a radius of 1 km or 10 km. We also include estimates of station-level variance $({{\rm{\omega }}}_{{p}_{s}})$ as for breeding density.

Our survival model was a hierarchical formulation of the transient Cormack-Jolly-Seber model^60^ and included encounter histories for *i* = 1, …, *N* individuals and *t* = 1, …, *T* sampling occasions where an occasion is equivalent to a year. Our modeling approach was similar to Saracco *et al*. (refs^20,55^) but without the spatial autoregressive structure since there were too few MAPS stations with sufficient numbers of Canada warblers to include this type of structure in our models. Survival analyses were based on MAPS stations with at least 5 marked individuals across all years resulting in 1203 marked individuals from 37 stations. This included 664 individuals from 18 stations in the western region, 191 individuals from 3 stations in the central region and 348 individuals from 16 stations in the eastern region. MAPS capture data typically include transient individuals that are only passing through the station area and not a part of the local breeding population. If we do not account for these individuals in the analysis, our estimates of apparent survival will be biased low. Therefore, our model included the estimation of apparent survival probability (ϕ) and the probability that a captured individual was a resident (π). We also estimated two nuisance parameters, recapture probability (*pr*), which is the probability of capturing an individual given that it is alive and in the study area, and the probability of pre-determining an individual as a resident (ρ). The latter can be estimated because some individuals were captured in more than one MAPS period each year and are therefore known to be residents. Further detail on the development of the model is provided as Supplemental Material (S1).

The apparent survival probability (ϕ) and residency probability (π) of individual *i* at time *t* was estimated with a common intercept (μ and α respectively), an influence of region $({{\rm{\beta }}}_{{\rm{\varphi }},{1}_{k}},\,{{\rm{\beta }}}_{{\rm{\pi }},{1}_{k}})$, variation by sex $({{\rm{\beta }}}_{{\rm{\varphi }},2},\,{{\rm{\beta }}}_{{\rm{\pi }},2})$, and in relation to human footprint at 1 km and 10 km radius scales around MAPS stations $({{\rm{\beta }}}_{{\rm{\varphi }},3},\,{{\rm{\beta }}}_{{\rm{\pi }},3})$. The two radii were included separately in models as for breeding density and relative breeding productivity. For apparent survival we also examined region-specific temporal trends $({{\rm{\beta }}}_{{\rm{\varphi }},{4}_{k}})$. Station-level variance (${{\rm{\omega }}}_{s}$) was included for both probabilities:

3 $${\rm{logit}}({{\rm{\varphi }}}_{i,t})={{\rm{\mu }}}_{\varphi }+{{\rm{\beta }}}_{{\rm{\varphi }},{1}_{k}}+{{\rm{\beta }}}_{{\rm{\varphi }},2}\ast {{\rm{sex}}}_{i}+{{\rm{\beta }}}_{{\rm{\varphi }},3}\ast {{\rm{HF}}}_{s}+{{\rm{\beta }}}_{{\rm{\varphi }},{4}_{k}}\ast t+{{\rm{\omega }}}_{{{\rm{\varphi }}}_{s}}$$

4 $${\rm{logit}}({{\rm{\pi }}}_{i,t})={{\rm{\alpha }}}_{{\rm{\pi }}}+{{\rm{\beta }}}_{{\rm{\pi }},{1}_{k}}+{{\rm{\beta }}}_{{\rm{\pi }},2}\ast {{\rm{sex}}}_{i}+{{\rm{\beta }}}_{{\rm{\pi }},3}\ast {{\rm{HF}}}_{s}+{{\rm{\omega }}}_{{{\rm{\pi }}}_{s}}$$

We also used logit-linear functions for the estimation of pre-determined residency (ρ) and recapture probability (*pr*). Both parameters were defined with a common intercept, an influence of sex and a station-level random effect (ω).

There were no year-to-year recaptures of individuals marked as juveniles and therefore we were unable to estimate juvenile survival using the approach described above for adults. However, because we can estimate region-specific trends in abundance and adult apparent survival we can estimate trends in recruitment as a derived parameter based on the following equation:

5 $${{\rm{rec}}}_{k,t+1}={N}_{k,t+1}-{N}_{k,t}\ast {\varphi }_{k,t}$$

where the number of new recruits at time *t* + 1 is equal to the region-specific index of breeding density at time *t* + 1 $({N}_{k,t+1})$ minus the region-specific index of breeding density at time t $({N}_{k,t})$ multiplied by breeding adult apparent survival from time *t* to $t+1({\varphi }_{k,t})$. As defined in this manner, the estimated number of new recruits represents the combination of juveniles that recruited in their second year plus adult immigrants. If there has been no directional change in adult emigration or immigration at all MAPS stations within a region, then this estimate represents an index of trends in new juvenile recruits to the population.

While we estimated change in demography over time using a linear trend parameter by region, an alternative model structure is to separately estimate the annual indices for each region as done for similar analyses on other species^20,55^. The estimation of annual indices requires a large number of parameters and a spatial autoregressive structure can be used to better estimate the indices. Canada warblers are relatively uncommon compared to many Neotropical migrants and we did not have the sample size of individuals nor the density of MAPS stations to reliably estimate annual indices using a spatial autoregressive approach.

### Model implementation

We fit all abundance and demographic models using Markov Chain Monte Carlo methods in JAGS implemented through R with package r2jags^61,62^. Productivity was estimated separately, while analyses for density and apparent survival were conducted within a single model, allowing us to estimate recruitment. By analyzing recruitment within the same model, the uncertainty in the estimates of abundance and apparent survival were incorporated into the recruitment estimate. For all response variables, we estimated beta parameters as random effects drawn from normal distributions with fixed effect means and variances. We used uninformative normal priors with mean 0 and variance 10^3^ for beta parameters. We specified the variance associated with station, year and noise as mean 0 random variables and used inverse-gamma prior distributions with shape and scale parameters = 0.001 for the variance estimate. For all analyses we ran two chains for 100,000 iterations, discarded the first 80,000 iterations as a burn-in and thinned by 2 to give 20,000 samples from the posterior distribution for inference. We assessed model convergence through the parameter history plots and R-hat convergence diagnostics^63^. We present the 90% highest posterior density interval as our credible intervals for all rates. In this case an upper interval for the beta coefficient below 0 indicates a 95% probability of a decline over time or a negative response to human footprint for the respective demographic parameter. We also report the proportion of the posterior mass that excludes 0.

### Data availability

The datasets generated during and/or analyzed during the current study are available from the corresponding author on reasonable request.

## Electronic supplementary material

Supplementary Information
