# Supplementary material for: Drivers of demographic decline across the annual cycle of a threatened migratory bird
Source: Sci Rep. 2018 May 9;8:7316. doi: 10.1038/s41598-018-25633-z (PMC5943453; doi:10.1038/s41598-018-25633-z)
Supplement: Supplementary file 1 — Supplementary Information [file 41598_2018_25633_MOESM1_ESM.docx]

**Supplemental Material**

Drivers of demographic decline across the annual cycle of a threatened migratory bird

Scott Wilson^a^, James F. Saracco^b^, Richard Krikun^c^, D. T. Tyler Flockhart^c,d^, Christine M. Godwin^e^ & Kenneth R. Foster^e^

^a^ Wildlife Research Division, Environment Canada, National Wildlife Research Centre, 1125 Colonel by Drive, Ottawa, ON, K1A 0H3, Canada

**^b^** The Institute for Bird Populations, PO Box 1346, Point Reyes Station, CA 94956

^c^ Lesser Slave Lake Bird Observatory, Box 1076, Slave Lake, AB, T0G2A0

^d^ University of Maryland Center for Environmental Science, Appalachian Laboratory, 301 Braddock Road, Frostburg, MD 21532

^e^ Owl Moon Environmental Inc., 324 Killdeer Way, Fort McMurray, Alberta, T9K 0R3, Canada

Corresponding author: Scott Wilson, email:scott.wilson@canada.ca, phone: 613-990-9740

**Supplemental Figure Legends**

**Figure S1.** Canada warbler distribution maps showing breeding range in North America and winter range in South America. Maps were created in QGIS Version 2.18^1^ using predicted distribution maps from eBird spatio-temporal exploratory models^2,3,4^. We selected 5-week periods for the breeding (June 6 – July 11) and wintering (Dec 26 – Jan 30) ranges and merged the weekly raster layers to create a single layer for the breeding and the winter range. Distribution maps are displayed on top of the 2009 human footprint index which was created using the raster layers in ref. 5. Darker colors on the map indicate areas of higher footprint (see also Fig. S2).

**Figure S2.** 2009 human footprint index^5^ for North America (top) and Central America and northern South America (bottom) with darker colors indicating areas of higher footprint. The figure was created using QGIS Version 2.18^1^ from the raster data provided in ref. 5.


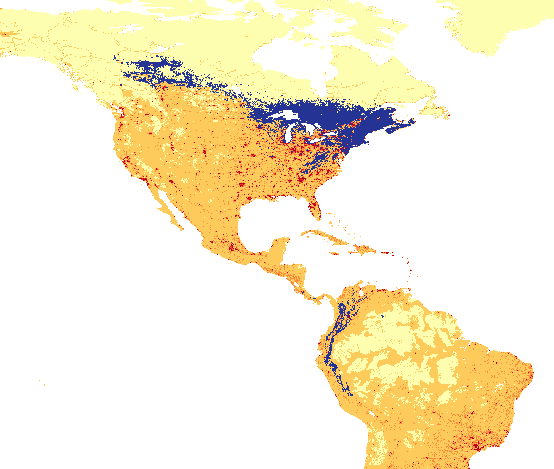


Figure S1.


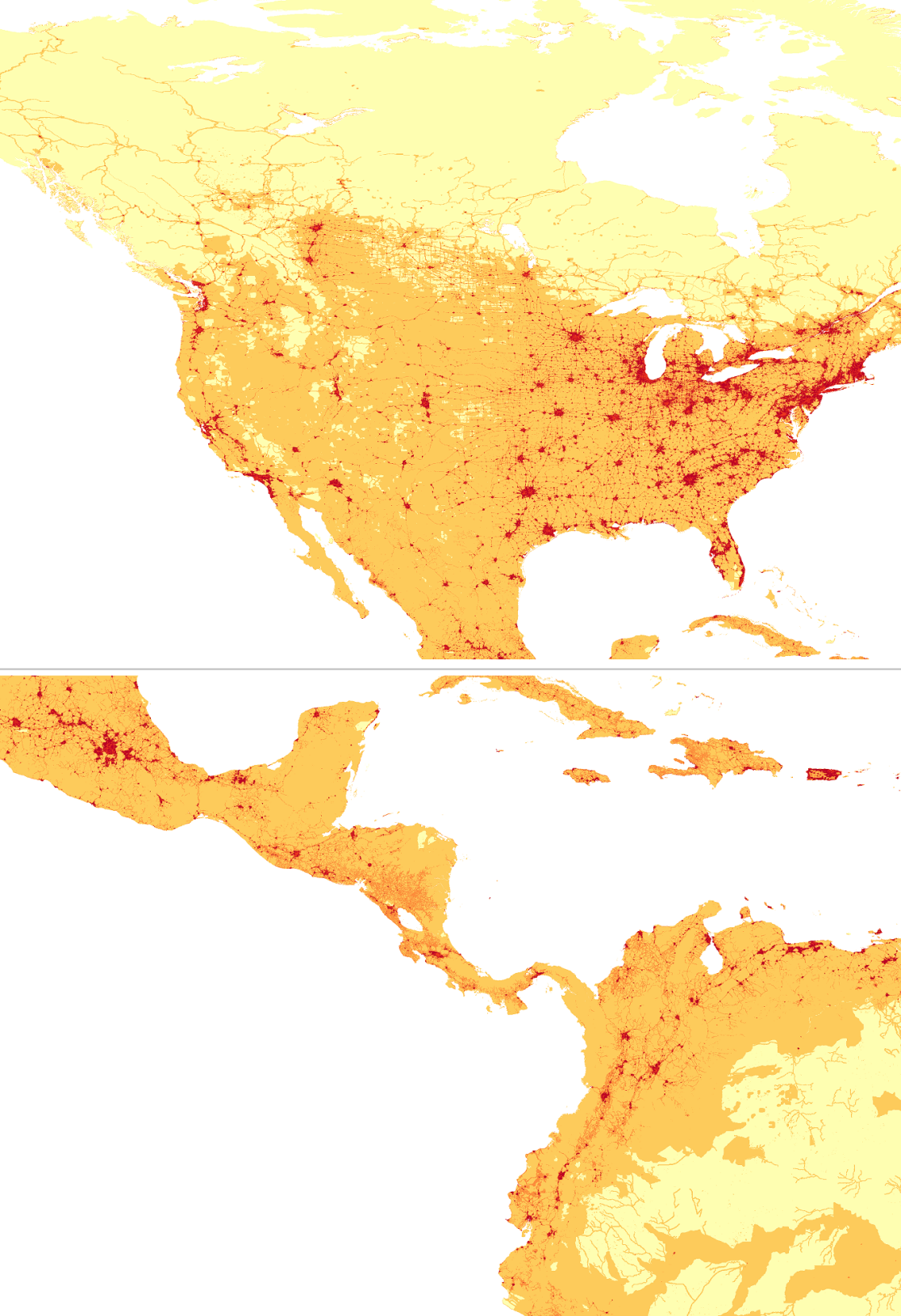


Figure S2.

Table S1. Trends in breeding abundance, productivity, apparent survival and recruitment for Canada warblers from western, central and eastern regions of the breeding range (see also Fig. 1). Values include the mean and 90% credible interval for the annual percent change in each rate; an upper interval below 0 indicates a 95% probability of a decline. For all four rates, the value in the second row is the probability of a decline based on the proportion of the posterior mass below 0.

|  | West | Central | East |
| --- | --- | --- | --- |
| Breeding abundance | -1.48 (-2.62, -0.32)  0.98 | 1.21 (-0.77, 3.33)  0.15 | -4.64 (-7.47, -1.83)  >0.99 |
| Productivity | 0.66 (-1.22, 2.52)  0.28 | 1.08 (-1.65, 3.74)  0.25 | 0.57 (-3.29, 4.40)  0.40 |
| Apparent survival | -0.22 (-3.39, 2.68)  0.54 | 6.35 (-0.74, 12.96)  0.06 | -2.74 (-11.87, 6.31)  0.70 |
| Recruitment | -1.39 (-2.89, 0.19)  0.92 | -1.46 (-5.18, 2.49)  0.74 | -3.66 (-7.75, 0.62)  0.91 |

**Supplemental Methods. Additional detail on the methodology used to estimate apparent survival and residency probability.**

Our model was developed with two latent state variables, residency state, *R*(*i,t*) and alive state, *z*(*i,t*), which represent the true residency and survival respectively for individual *i* in year *t*. Conditional on the true states we have two observed variables from the data, predetermined residency, *r*(*i,t*) and observed alive *y*(*i,t*). We are unable to directly observe the true residency state of an individual. However, because MAPS data are collected over multiple periods within a year we can pre-determine some individuals as residents in the data set when they are captured in more than one MAPS period. These pre-determined individuals were identified with an indicator, *r*(*i,t*), such that *r*(*i,t*) =1 for a pre-determined resident and *r*(*i,t*) = 0 for all other individuals. The observed residency is then expressed as a Bernoulli random variable conditional on true residency:

*r*(*i,t*)| *R*(*i,t*) ~ Bern[*R*(*i,t*), ρ*_i,fi_*]

where ρ*_i,fi_* is the probability of pre-determining an individual as a resident. In the estimate of pre-determined residency probability, ρ*_i,fi_*, we use subscript *fi* to note the first year when the individual was marked as this is the period when residency is determined. The true residency of individual *i* is also defined as a Bernoulli random variable:

*R*(*i*) ~ Bern (π*_i,fi_*)

where π*_i,fi_* is the probability that individual *i* at time *fi* is a resident. Under this formulation, the probability that a non-resident [*R*(*i,t*) = 0] is identified as a true resident [*R*(*i,t*) = 1] is 0 while the probability that a true resident [*R*(*i,t*) = 1] is defined as a pre-determined resident [*r*(*i,t*) = 1] is ρ*_i,fi_*. The identification of residency probability permits a more reliable estimate of survival and it is partially a nuisance parameter to account for transiency, although variation in residency probability is also of interest in an ecological context. In our data, 182 of 1203 (15.1%) banded Canada warblers were pre-determined as residents.

The survival process is described by an individual’s true “alive” state *z*(*i,t*) where *z*(*i,t*) = 1 for individuals that are alive and in the sampling area during time period *t*, and *z*(*i,t*) = 0 for individuals that are dead or not yet banded. The true alive state is then defined as:

*z*(*i,t*)| *z*(*i,t*-1) ~ Bern[*R*(*i,t*) *z*(*i,t*-1) ϕ*_i,t_*_-1_]

where a resident individual’s alive state at time *t*, *z*(*i,t*), given that it was alive during the previous time interval, *z*(*i*,*t*-1), is a Bernoulli random variable with survival probability ϕ*_i,t_*_-1_. Individuals that were not residents [*R*(*i,t*) = 0] or were not alive and in the study area at time t-1 [*z*(*i,t*-1) = 0] will have an alive state *z*(*i,t*) at time *t* = 0.

We cannot directly observe an individual’s true alive state and therefore it was modeled based on the observed encounter histories:

*y*(*i,t*)| *z*(*i,t*) ~ Bern[*z*(*i,t*), *pr_i,t_*]

which states that conditional on individual *i* being alive and in the sampling area at time *t*, we can observe that individual with recapture probability *pr_i,t_*. The estimation of recapture probability is derived from the encounter histories when an individual is missed during a primary sampling occasion (i.e. not detected in a year). Although our model estimates transiency, we are unable to account for individuals who are identified as residents in one year but subsequently emigrate out of the study area. Therefore, our estimates of survival are apparent survival and equal to the product of true survival and fidelity to the area of each MAPS station.

**Supplemental References**

1. QGIS Development Team. QGIS Geographic Information System. Open Source Geospatial Foundation. URL <http://qgis.osgeo.org> (2009).
2. Fink, D. *et al.* Spatiotemporal exploratory models for broad-scale survey data. *Ecol. Appl.* **20**, 2131–2147 (2010).
3. Johnston, A. *et al.* Abundance models improve spatial and temporal prioritization of conservation resources. *Ecol. Appl.* **25**, 1749–1756 (2015).
4. Fink, D. *et al.* Modeling avian full annual cycle distribution and population trends with citizen science data. *bioRxiv* 251868. <https://doi.org/10.1101/251868> (2018).
5. Venter, O. *et al*. Sixteen years of change in the global terrestrial human footprint and implications for biodiversity conservation. *Nat. Commun*. **7**, 12558 (2016).
